# Case of a Fractured Scull Unsuccessfully Treated

**Published:** 1789

**Authors:** John Grimston

**Affiliations:** Surgeon at Ripon


IV. *Cafe of a fractured Scull unfuccefsfully treated. By Mr.* John Grimfton, *Surgeon at Ripon. Communicated to Dr.* Simmons *by Mr.* Lucas, *Surgeon at Leeds.*

ON the 13th of October, 1788, T. Wilfon, a boy four years old, was knocked down by the heel of a horfe, and wounded on the right fide of his head. By enlarging the wound, I difcovered a fracture of the right parietal bone, croffing the coronal future and extending to the frontal bone. The fracture, which was three inches and a quarter in length, and an inch and a half in breadth, was attended with confiderable depreffion.

By the immediate application of the trephine I was enabled to remove all the depreffed pieces of bone, which I found had wounded the meninges, fo as to occafion portions of the brain to be forced out of the wound.

Such diet and evacuations as were occafionally indicated, were ftrictly enjoined, by which means pain and fever were, in a great meafure, prevented.

A few days after the operation the aperture in the bones was filled with a fungous fubftance,

ſtance, of rapid growth, which was gradually reduced by a ſolution of blue vitriol and preſſure; and for a month or ſix weeks the health of the patient and the appearance of the wound afforded the greateſt proſpect of recovery.

In the early part of December there again appeared, in the anterior part of the fracture, a hard tumour, of about the ſize of a pea, which increaſed greatly, notwithſtanding various attempts to reſtrain it were uſed, both by preſſure and cauſtics, as recommended by different authors*. I was therefore induced to remove it (as near its baſe as poſſible) by ligature, which was effected with hardly any pain to the patient; and then the compreſſion was aſſiduouſly repeated.

In the beginning of February, 1789, I firſt obſerved, that, when my patient laughed, his mouth was drawn to the right ſide; but after the diſcharge of even a ſmall quantity of pus, from any part of the opening in the bones, this affection conſtantly diſappeared.

On the 23d of February, after he had eaten his dinner, and been playing with his uſual cheerfulneſs, he began ſuddenly to complain of

* Gooch, Warner, Bell, &c.

ficknefs, which was fucceeded by vomiting and fever, (from which he had continued hitherto free); his left arm became convulfed; his mouth and eyes were feized with almoft perpetual involuntary motions, yet the pupils of his eyes were not uncommonly dilated, nor did his fight feem impaired; but he was totally infenfible.

As it was fufpected that thefe fymptoms might arife from a detention of matter within or under the fungous eminence, which had again protruded as much as ever, a deep incifion was made through the tumour until about an ounce of pus was evacuated; after which an evident abatement of the fymptoms took place, but within an hour they returned with increafed violence.

By means of the application of leeches, and the ufe of cathartics occafionally, we fo far fucceeded, that the fymptoms of preffure on the brain returned only at intervals, and were never violent until the 11th of March. On that day he became infenfible for an hour, and Dr. Harrifon, a phyfician of this town, having been confulted, he agreed with me in opinion that a fimilar attempt fhould be made as before to to difcharge any matter that might be collected within the fcull.

A large quantity of colourlefs fluid was accordingly evacuated, and this difcharge afforded confiderable relief to the patient. On the following day the whole of the prominence was removed by incifion, but no more fluid appeared to be confined by it. The texture of the protruding fubftance varied much; at one time refembling the brain, at another cartilage, or fteatoma.

After this the tumour foon began to rife again, and increafed with the fame rapidity as before, nor would it bear much preffure. Vomiting, with a variety of fpafmodic affections, efpecially a rigidity of the neck, to which the patient had not before been fubject, occurred now very frequently.

Although no fluid could be difcharged from the tumour, which became gradually flaccid, and much diminifhed, yet the fymptoms fo far continued as to reduce him quickly, and on the 31ft of March he died.

On examining the brain after death, it appeared that two wounds in the dura mater, inflicted at the time of the accident, continued open, and that the tumour had its origin from that that membrane, to which it inseparably adhered. An empty cavity, large enough to have contained seven or eight ounces of fluid, had been formed by the pressure of the tumour. As every other part of the brain seemed to be perfectly sound, the death of the patient might, I think, fairly be attributed to this pressure upon the brain.

The continuance of the disease without alarming symptoms; the prospect we so long had of the patient's recovery; the resemblance of the symptoms he laboured under to those produced by paralytic affections; and lastly, the inefficacy of the means employed for suppressing the fungus, are all of them circumstances that appear to merit attention.

Perhaps there are no cases attended with so much uncertainty in their event as those which arise from injury done to the contents of the cranium; and hence it is difficult to ascertain the time when danger is over, or even when the utmost caution in the treatment of the patient may be relaxed.

The sudden and violent manner in which this little patient was seized after a full repast, seemed to resemble much what happens in paralysis, ralyfis, and may perhaps lead to a farther inveftigation of the caufes of that difeafe.

With refpect to our endeavours to fupprefs the fungus, I cannot avoid having my doubts whether cauftic applications anfwer in fuch cafes; or whether even preffure does good, unlefs applied early, and continued for fome time, efpecially as thefe tumours often fubfide of themfelves.

The good effects, however, of compreffing a threatening protuberance are founded upon the beft authority, and I have no doubt have been fully experienced in many cafes.

The treatment of this fungous fubftance feems of more importance than many authors have imagined, as to the failure of its fuppreffion may be attributed the death of my patient.

It was in agitation to have repeated the application of the trephine at the pofterior part of the fracture, as from that part the difcharge feemed to arife; but I am inclined to think that a repetition of the operation there would have anfwered but little purpofe, as the difeafe appeared to be confined to the anterior part.

The unpleafing tafk of defcribing a failure in practice has perhaps deterred many from

 commucommunicating facts which might have tended to throw light not only on the grounds of the failure, but also on the cause of the symptoms and on the means of future success.

"It is to be wished," observe the learned Editors of the Medical Transactions *, "that "writers would not confine themselves to re- "late only their successful practice. A physi- "cian of great experience might write a very "useful paper if he would have the courage "to give an account of such methods of cure "only as he had found to be ineffectual or "hurtful."

